# Assessment of a Workforce Sustainability Tool through Leadership and Digitalization

**DOI:** 10.3390/ijerph20021360

**Published:** 2023-01-11

**Authors:** Ioana Gutu, Daniela Tatiana Agheorghiesei, Alexandru Tugui

**Affiliations:** Faculty of Economics and Business Administration, Alexandru Ioan Cuza of Iasi, 11 Carol I Boulevard, 700506 Iasi, Romania

**Keywords:** workforce sustainability tool, leadership, digital learning

## Abstract

As organizational environment develops, the working environment increases in physical and mental demands. As a result, risk inadvertences could arise, along with organizational emotional and financial challenges. Within their efforts to diminish such risks, organizations strive for developing and training their workforce; a sustainable workforce can only be achieved through cultivating aptitudes and positive attitudes that will lead to organizational but also personal growth. Considered to be an important measuring instrument for social sustainability, workforce sustainability enhances organizational leadership projections and trajectories, along with digitalization initiatives. The aim of the current study is the development of an assessment tool for state and private organizational workforce sustainability, and to study it in relation to leadership and digitalization components. Through a quantitative approach, data was gathered by issuing an online survey that delivered 463 responses. By using structural equation modelling, the authors examined the aims and found that the designed workforce sustainability tool is reliable and valid; as predicted, all the leadership components contribute to organizational stability and a more favorable workforce sustainability development, along with enhancing digital learning. This study stresses the importance for state and private organizations to achieve workforce sustainability, while nurturing and providing the necessary tools for the development of leadership and digital learning.

## 1. Introduction 

In line with the practices of the unique nature of organizational growth, a high level of organizational sustainability is being implied; altogether, continuous efforts for improving equity and value are being undertaken. The sustainable development of specific industries (e.g., health, IT, chemical, financial banking and or/biology) is expected to reinforce the well-being of the workforce and the maturity of the organizations. For a specific industry to subscribe to sustainable standards, social sustainability must be rated as high priority in terms of education, economic welfare and knowledge and skill diversity. 

Sustainable development expresses a holistic view that addresses economy, society, environment and common practices that need to be shared in order to be adopted and uniformly implemented across industries. As most of the rating systems concur with the internal standards for reporting, practices for implementing sustainability are not standardized, and therefore, the process is not complete. To respond to this deficiency, workforce sustainability must be clearly defined, measured and time-bounded [1].

Based on a thorough literature review, a prior study that provided a model for assessing a company’s workforce sustainability framework was not found; the few studies that regarded workforce sustainability did not surpass the theoretical boundaries. Workforce sustainability represents a basic concept scarcely approached by literature [2,3,4]; previous results gather a different number of components and study angles, without reaching to a complete comprehensive result.

The current study intends to create a theoretical and practice bridge, by developing and validating at first, a practical model designed to assess organizational workforce sustainability, and further analyze the connection between workforce sustainability through its eight dimensions on one side, and the transformational leadership and two specific components of transactional leadership, along with a specific component of digitalization, as a tool for organizational learning and education. 

The study was initiated by performing a fully comprehensive literature review, by following the rules specific to the systematic review (SR) approach, where a review of both online library and multi-industry resources was included, thus allowing a complete examination of publications in regard to the studied subject. Following this perspective, the research included a quantitative approach, where workforce sustainability and related concepts were analyzed as latent variable modelling by using the SmartPLS software (v. 4.0.0).

Researchers need to emphasize that, for the purpose of this study, workforce specific to a wide range of industries was considered. 

Furthermore, the current approach focuses on workforce sustainability leadership and digitalization in its natural context, thus reducing recall bias since data is collected from active organizational workers. By adopting an organizational perspective, the research provides a more comprehensive framework of a sustainable workforce environment, where the perspective of applied leadership and digitalization instruments play important roles. 

The present study contributes to the existing literature by examining, first, the unique contribution of designing and assessing a research instrument that defines all the attributes of the workforce sustainability, along with creating a structural model that explains the relationship that regards the eight dimensions of the construct. Previous research has shown the importance of leadership instruments in assessing and enhancing desired workforce behaviors [5,6]; but it appears that literature has ignored the role of leadership (as transformational and transactional) on workforce sustainability. We argue that leadership components such as transformational leadership, management by exception—active and contingent rewards are effective in stimulating a favorable workforce organizational environment. Moreover, studying the underlying process specific for applying digital tools by followers within the learning processes, provides us with a more advanced understanding of workforce sustainability, leadership and digitalization. 

### 1.1. Theoretical Background 

Along with terms such as stakeholder and social responsibility [7], popularized across literature and business practice, the term, sustainability, was introduced [8]. Another perspective introduces the Brundtland report as staying at the origin of the concept, where the definition of sustainable development stressed over “meeting the needs of the present, without compromising the ability for future generations to meet their own needs” [9]. Reporting on the implementation, management and promotion of corporate responsibility [10] became of large interest for companies across the world, who adopted and included within the internal specific standards actions that would make the organization socially responsible [11]. Global trends in regard to corporate sustainability reported that by 2020, 80% of worldwide companies woyld issue annual reports in regard to sustainability, while GRI continues to be considered the dominant global standard [12] for sustainability reporting. Among the most prioritized sustainable development goals were those that reported on productive employment and decent work for all (72%) and innovation (50%); the same report states that companies increased the reporting of sustainable development goals to 14% in the previous reporting year. It is considered [13] that the trend in sustainability reporting is a consequence of pressures coming from regulatory policies [14] and stakeholders [15]. A second perspective emphasizes companies proactivity for increasing productivity, lowering cost production and enhancing brand equity. A third opinion introduces into the sustainability trend-line the ethics as a manifestation of top and middle management and founders [16]. From a skeptical point of view [17,18,19,20], companies annual reporting in regard to state and private organizational sustainability performance may be shadowed since there is a high probability that management systems for measuring performance (may) have not been implemented.

Both transformational and transactional leaders act for enhancing their followers’ motivation [21], in order to increase their effectiveness and perform over expected results. Since leaders do not use only one of the two leadership styles, but weave among both, research has shown that one of the two styles prevails, namely the transformational leadership style. According to other studies [22], the two leadership styles have the ability of predicting different outcomes, which on an organizational level may be important predictors for the sustainability of the workforce within a given environment and/or industry [23]. If taking in consideration the medical industries across the world, data has shown that traditional approaches with regard to workforce management and planning is no longer suitable, since the working conditions are directly subjected to an ever increasing number of public-private-NGO partnerships who rely on policy dialogues when considering the sustainability of the workforce, but do not have any proper assessment instruments to create organizational inner analysis and industry outer analysis [24]. Despite the increasing importance of social interactions, leaders have adapted their tools and instruments for guiding their followers not only in a physical but also within a virtual world, where digitalization plays a key role. While teaching and coordinating working groups, the use of digital instruments in order to fulfill workforce teaching and learning expectations prove to be necessary for learning organizations. 

### 1.2. Sustainability on an Organizational Level

Across political arenas, academia, press and corporate social and business events, (public or private organizational) sustainability counts across the prominent topics to be discussed. Originating from the general management literature [25] (corporate) sustainable development was defined at first in 2005 [16] as a tridimensional construct that included not only environmental integrity, but also social equity and economic prosperity. There is little insight about the interaction process and consequences between the cultural orientation specific to a business and the corporate sustainability principles [26]. At its core, sustainability assumes a range of movements across time that include conservationist, environmental and technological, followed by the no-growth philosophy. Further, ecology and social movements (such as human rights, poverty and life quality) redesign the concept of sustainability. 

In 1987, the World Commission on Environment and Development (WCED), also known as the Brundtland Commission [27], issued a report (Our Common Future) that related to social equity and environmental integrity and defined a new term, sustainable development; a corporate assessment of the newly emerged concept of sustainable development integrating (strategic) management and achievement of organizational goals [28]. There are three main views that relate to the term of corporate sustainability: ecological responsibility; the social responsibility of a corporation; and the natural and social environment are considered within corporate economic activities [29,30]. As previously defined, corporate sustainability is known under the title of sustainability for business [31], that included several values such as prestige and business reputation, product and services quality, stakeholder added value and ethical business processes. Further, authors assumed that corporate sustainability is similar to the corporate social responsibility concept and consider that a corporation should include within its economic, strategic, operational and/or cultural decision-making elements, such as economic, social or environmental issues [32]. 

Finally, it appears to be difficult to retain a definition that clearly defines corporate sustainability, reason for which corporate members find the assimilation process of the practices assumed by this term to be confusing and hard to implement [33]; researchers emphasize the lack of procedural clarity when implementing corporate sustainability within the internal procedures of an organization [34]. 

It is important to notice that the first literature assessments in regard to corporate sustainability implementation focused on the external factors of the organizations; for this reason, special attention was given to sustainability practices and classifications [35,36,37]. The process suffered alterations over time, so that special internal pressures within organizations (such as staff turnover, management support, employee empowerment, workplace satisfaction) gained visibility [38] and started to be considered as important steps for achieving corporate sustainability. Certain approaches [39] suggest that corporate sustainability resides more within underlying assumptions that changing employee values and behaviors are the real factors for companies to focus on [40]. This wide range of (dis)agreement with regard to corporate sustainability practices and implementation recipes suggest only that this concept is multifaceted [41] and requires different organizational levels of adaptation. 

Following the current perspective, an organizational characterization that emphasizes the adoption of three necessary levels of actions for a corporation in order to be sustainable can be retained; these views accompany the temporal evolution of the concept and start with the underlying level [40], which emphasizes the role of interdependence of human and ecological factors as necessary principles specific to corporate sustainability; the value level as previously defined [42], brings special attention to employee values and beliefs that need guidance toward high levels of ethics and social responsibility; the generally perceived level (as the surface level with unconscious actions and beliefs) pays special attention to technical human resources departments, responsible for assessing employee performance and/or training and issuing internal corporate sustainability reports.

### 1.3. Workforce Sustainability—A New Concept Defined within the Literature

The added value of the literature on workforce sustainability suggests that as derived from corporate sustainability, workforce sustainability is a concept that is considered to be new to most of the industries; from this perspective, Romania’s industry is no exception. An organization is considered to be workforce sustainable when high levels of productivity are achieved, and personnel declare they are satisfied with regard to the work environment they act within. Further back, the concept was considered to be a result of work policies and procedures that enabled two main points of view: employment practices and personnel family lives [43]. Therefore, for a company to achieve workforce sustainability [44] it is necessary to create at first an internal environment that supports competent, motivated and coherent individuals, followed by a long-term strategy that will not only enable but also nurture workforce competencies [45]; for this reason, training, personal and career development strategies will be needed.

As for this perspective, workforce sustainability develops as a concept that reflects an internal Human Resources (HR) business design that must comply with the task of assuring the workforce performance [1] and functioning over a given time schedule. This specific HR task will grant the workforce with the ability of developing its own safe, equal and connected work environment, where values such as education, honor, equity and equality will be supported; moreover, businesses could create and enhance workforce sustainability infrastructures that could transform and become self-sustaining over time. 

It is symptomatic to observe that academic standards need to be applied for the current study, where workforce sustainability will be analyzed by using a Systematic Review (SR) and assess different type of evidence, further enclosed. 

### 1.4. Research Flow 

Research assumed that literature provides reliable data with regard to the assessment and development of a tool providing state and private organizational workforce sustainability. In order to better understand the state-of-the-art in regard to such endeavors, researchers considered that there will not be a better fit than a procedure that considers the rules applicable to a systematic review (SR), as a widespread method considered in order to summarize accurate and reliable data from different type of evidence (article, book chapters, reviews etc.). According to literature, the bias of this method is that it is directly connected to how well the authors reported particular data important for the performed study. 

As for retrieving the relevant datasets in regard with previous literature developments of an assessment tool for workforce sustainability, the Scopus database (https://www.scopus.com/ (accessed on 2 November 2022)) was considered; the search was conducted by using the Title, Abstract and Keywords as the key-aspects of the selected items. The search strategy was developed within the interval 01–22 November 2022 and included all the articles that subscribed to the researched topic.

Except for “workforce sustainability” terminology, the study also included the term “sustainable workforce” since it is commonly used when referring to “workforce sustainability”. To subscribe to the complexity of the audience definitions, the Boolean logic algebra form was considered suitable, therefore the search included operators such as “Or”, “And”. By complying with the abovementioned conditions, the search string was shaped as “Workforce sustainability” OR “Sustainable Workforce” AND “Tool”. By performing the search, data retrieved showed 17 document results to be considered at first. 

Since the study does not involve any patient or human implications, no ethical approval was considered as necessary for drafting the current study. 

As for the search strategy, in order to refine the results, the online library’s eligibility criteria were used, in order to screen out irrelevant studies that do not answer the current study’s research questions. The criteria assume both inclusion and exclusion conditions for the studies to be met. As for the current case, the common rules presume two limitations (exclusion criteria as EC) that could be applied. 

EC 1. The language limitation: all the documents should be written in English (therefore excluding articles published by using other languages). The authors considered that any language barrier should be removed, since a large (r) group of readers should be considered when referring to the audience’s wideness. 

EC 2. Limitation to scientific interest have been applied to exclude bibliometric analysis from the current search. The reason behind the current choice is that bibliometric analysis is considered to be a widespread and therefore popular method that is generally used for exploring large data volumes.

No specific limitation in regard to date range, source type (as qualitative or quantitative methodological approach), the document type (as book chapter or article), nor whether the data was open access, was taken into account.

The results show an initial collection of 17 titles that comply with the specified eligibility criteria. Since the systematic “workforce sustainability” and “sustainable workforce” “tool” studies are complete, the assessment of the included studies was in order. The Quality Assessment (QA) of the selected studies was considered, where the abstract and titles are considered as quality criteria, and thus defining the consistency of the current review in regard with the defined purpose of the selected items. To avoid bias as favoring one item from another, two of the authors screened the eight titles by working independently, and by using a prespecified research plan. No result difference was recorded. As part of the quality assessment, a range of checklist reasons was raised: (1) whether for the selected items, the full list of authors, abstract and keywords were provided; (2) the study aim and objectives were clearly stated; (3) the research methodology was explicitly provided within the study; (4) the study’s findings are clear and relevant for the specific titles; (5) the “workforce sustainability” dimensions and items were fully disclosed; and (6) the subject of workforce sustainability was clearly assessed, correlating with the theme of the selected item. 

According to previous literature [46], when an analysis considers a bibliometric analysis, according to the SR search flow, a first action should regard selecting and extracting the relevant studies for the current research. The results show that out of 17 primary results, only one item was considered to meet the eligibility criteria (as title and relevant abstract) and was further analyzed.

The one title that was retained from the data considers the development of an assessment tool that entirely refers to workforce sustainability [4]. Within their study, the authors determined the workforce sustainability attributes and performed an analysis as to assess the influence level when achieving workforce sustainability; moreover, they determined the indicators and metrics needed to be used for assessing the identified workforce sustainability attributes. 

It is also important to address the question of whether there are only two scientific inquiries which developed the only two complementary studies that consider the idea of defining workforce sustainability through its attributes [1,4]. For this reason, since the review of the Scopus database only conferred a small number of available titles (one) with regard to workforce sustainability, a review of multi-industry resources was included, thus allowing an examination of publications with regard to the studied subject [47].

A short analysis of industries and subject area reports brings into light numerous other authors that tried to define workforce sustainability through several of its attributes [20,43,48] and others, as Table 1 will reveal. Previous research recognized within an evaluation the importance of four of the considered attributes (such as nurturing, equity, safety and diversity) when assessing the organizational workplace [49]. Moreover, the International Living Future Institute that administers the JUST label [50] can also be added to the list, since the rank they performed in determining people’s happiness also include the employment role within the given equation; moreover, they add nurturing, diversity and equity as having a key role when considering employees happiness at work. Another report developed in 2017 [23] referred to the “State of the American Workplace” and considers nurturing, connectivity, value and maturity as key components recognized by workers that sustain their employment. 

In order to perform a thorough analysis of literature, except for the keyworks used when following the rules for the SR, the authors also considered titles that refer to social sustainability as the initial concept that encloses workforce sustainability. The results show two more academic publications that relate to the abovementioned subject that refer to workforce sustainability attributes; therefore, when another authors performed a study as referring to determining social sustainability criteria, such as for building new energy alignment projects, six attributes were identified and considered to be specific for workforce sustainability, such as nurturing, equity, connectivity, community, value and health and wellbeing. By analyzing the subject of highway construction [51] we identified eight similar attributes as earlier proposed [52]; additional to the current titles, in 2014 a report included all eight attributes when developing a study regarding organizational strategies that are favorable for achieving work-life balance [53].

**Table 1:** Literature review results in regard to the development of the WS tool framework.

Reference Title and Authors List	Number of WS Attributes Considered for the Construct Design	A Different Attributes Identification
[4]	Nurturing, Diversity, Equity, Health and Wellbeing, Connectivity Community, Maturity	
[2]	Nurturing, Diversity, Equity, Health and Wellbeing, Connectivity, Value, Community, Maturity	
[3]	Nurturing, Equity, Health and Wellbeing, Connectivity Community	
[49]	Diversity, Equity, Health and Wellbeing, Connectivity, Value, Community	Nurturing = education Value = employee benefits;
[50]	Nurturing, Diversity, Equity, Health and Wellbeing, Community	Connectivity = sharing;
[23]	Nurturing, Value	Connectivity = employee engagement Maturity = employee performance
[32]	Nurturing, Diversity, Health and Wellbeing, Community	Maturity = ethics
[48]	Nurturing, Equity, Health and Wellbeing, Community	Value = wages
[54]	Diversity, Equity, Health and Wellbeing, Value, Community	Nurturing = awareness Maturity = leadership
[43]	Nurturing, Diversity, Health and Wellbeing, Connectivity, Community	Value- = compensation Maturity = knowledge sharing
[55]	Nurturing, Equity, Health and Wellbeing, Value	Maturity = ethics
[56]	Nurturing, Diversity, Equity, Health and Wellbeing, Value, Community, Maturity	
[57]	Diversity, Equity, Health and Wellbeing	Nurturing = training Value = wages and welfare
[58]	Diversity, Equity, Connectivity, Value, Community	Connectivity = engagement
[59]	Nurturing, Diversity, Health and Wellbeing, Value	Connectivity = engagement
[60]	Nurturing, Diversity, Equity, Health and Wellbeing, Connectivity, Value	
[61]	Nurturing, Value	Connectivity = employee engagement
[62]	Nurturing, Diversity, Equity, Health and Wellbeing, Value	
[63]	Nurturing, Health and Wellbeing, Community	Maturity = skills development

Source: adapted after [2], 2019.

The development of a workforce sustainability assessment tool, as literature suggests, can only begin by defining each of the eight identified attributes. Nurturing provides information related to the extent to which members of an organization are subject to productive performance appraisals, onboarding processes and technical skill training. Diversity, as the second workforce sustainability attribute, relates to ethnic, racial, gender, leadership diversity along with labor force and corporate policy inclusion. The third attribute, equity, could be used as indicator for the extent to which organizations allow equitable pay when considering both industry and organization levels, merit-based promotion plans and nondiscrimination. Through the health and wellbeing workforce sustainability attribute, organizations are encouraged to succeed for reaching zero injury goals, to include safety and health programs, to favor/allow social interaction at work and perform the legally recommended annual medical and/or physical checkups. Connectivity, as the fifth workforce sustainability attribute, analyses the extent to which personnel is included within decision-making and invited to one-on-one meetings with leaders/managers/supervisors, but also regards the social connectivity and organizational team approaches. The social extent of organizational actions that enclose social events and workforce integration, along with considering the local community needs and actions and the employee’s workload, constitutes the community attribute of the workforce sustainability concept. Value indicates policies in regard to full/partial time employment, insurances and retirement plans, work and family balance, along with benefit programs, appreciation and feedback at work. The eighth workforce sustainability component is maturity, and refers to the extent to which the organization fosters leadership skills and sets accountability standards among its workers, provides a framework for competence-based education and training and appreciates and includes internal and external volunteering.

**H1.**  *Workforce sustainability is positively related to the eight identified attributes as nurturing, diversity, equity, health and wellbeing, community, connectivity, maturity and value*.

### 1.5. Leadership–Transformational and Transactional

As previously considered [64], both transformational and transactional leaders motivate followers to reach and/or perform beyond already defined expectations. On an effectiveness scale, despite the fact that transactional leaders comply with expectations, transactional leaders promote the follower job performance exceeding the standard scale. Each leader performs by using extents of both leadership styles, but the most effective leaders tilt towards transformational leadership [65]. 

With regards to transactional leadership, it is argued that within an organizational context the workforce may be motivated by leaders [66]. Leadership, therefore, can be analyzed through its two main outcomes, also known in the literature as transformational and/or transactional, each of them having the ability to make a difference when motivating and influencing workforce attitude and motivation. The main idea in regard to transactional leadership resides within the fields of psychology and sociology [66]. The current idea was further adopted by literature [21,67,68,69], which agreed that transactional leadership primarily regards the reciprocal relationship between the leader and subordinate. The deterministic relationship as described above utilizes the bargaining process specific to a positively valued behavior.

As literature suggests [21,68], the transactional leader, in order to reach the desired behavior, sets patterns for (im)(ex)plicit patterns for reaching goals, by using contingent rewards (such as time off or paid supplementary work). Moreover, a transactional leader uses management by exception (active and passive) for implementing programs that will guarantee the desired behavior from the employee [65]. A transactional leader manifests a passive attitude and only reacts when forced by arising problems [69]. Management by exception (MBE) actively regards both leader and follower mistakes, anticipation and prevention [67]. On the opposite pole, MBE passive only considers manifesting disapproval and follower confrontation with their mistakes. Since MBE passive is specific to organizational contexts when leaders manifest intense control behaviors, for the development of the proposed research, only MBE active was considered. 

Comprising four dimensions (Idealized Influence—II, Inspirational Motivation—IM, Individual Consideration—IC and Intellectual Stimulation—IS), transformational leadership is generally used for follower motivation in order to obtain the leaders’ expectations. As for the II dimension, the items suggest that leaders are trusted and respected by employees, who perform as mirroring their behaviors. IM brings optimism into the equation and a positive vision of the future. Through IC, each follower’s need and ability is individually considered and acknowledged by the leader. Moreover, different perspectives for ideas and/or problems are considered through the IS dimension. 

Literature often compares transactional and transformational leadership within organizational contexts, the latter being universally recognized as highly inspirational, often referred to as the promotor of moral organizational developments across the entire workforce [21,68,70]. Transformational leadership uses as an instrument a facilitative power that allows leaders and managers together to prioritize and implement second-order changes across different departmental and educational arrays of workforce.

There were two authors that performed a meta-analysis regarding different arising perspectives and outcomes from practicing transformational and transactional leadership [69]. The results show that several variables (such as motivation, job performance) were predicted by transformational leadership and contingent reward (CR) as a component of transactional leadership; when MBE active, the impact on several outcomes variables was positive, but small. 

### 1.6. Workforce Sustainability and Leadership

Leaders’ endeavors with regard to (workforce) sustainability can be leveraged by implementing comprehensive sustainability strategies, by taking into account models of employee engagement [71] and transformational leadership practices [70]. The sustainability of the organizational workforce resides in efforts to hinger on organizational factors on a micro and macro level, resulting in a workforce sustainable performance, on both individual and/or organizational level. Therefore, business/organizational management strategies that lead to a workforce sustainable environment reside into leadership actions (MBE active) as examining and developing sustainable solutions that comply with the standards and the norms of a business environment, along with rewarding and appreciating followers (CR) according to their task fulfillment.

Within organizations, leaders are considered to be directly responsible in regard to planning, funding and workforce development. In order to achieve workforce sustainability, leaders need to initiate and grow change, by following the directions given by the policy makers and/or stakeholders [72]. 

Workforce sustainability is mainly subject to being achieved when leadership provides and favors the development of formal mentoring and the establishment of stronger internal and external networks and partnerships. Moreover, leadership is considered to be a workforce sustainability driver for clarifying issues in regard to remuneration and turnover, but also with opportunities to be achieved and infused by followers. Leadership has an essential role in providing staff with the appropriate working conditions and job satisfaction, recruiting and training staff with no/less qualifications, employee retention and maternity leaves and other benefits. 

According to literature [73], workforce sustainability is heavily influenced by management practices, such as recruitment, remuneration, the workers ability to communicate while at work (community) and use resources. A sustainable health workforce framework ca be defined by an organization with leadership that foster leader-follower relationships, access to internal and external resources is performed under an equity umbrella and favors workforce professional development. The current arguments lead to the following hypotheses:

**H2a.**  *Workforce sustainability is positively related to transformational leadership*.

**H2b.**  *Workforce sustainability is positively related to MBE active*.

**H2c.**  *Workforce sustainability is positively related to Contingent Rewards*.

**H2d.**  *The relationship between workforce sustainability and each of its eight attributes is moderated by transformational leadership*.

### 1.7. Digitalization through Learning and Expectations

Digitalization, as deriving from the Latin word *digitus*, provides information in relation to categories, integrity, countability and discretion in value and time. According to precedent approaches [74], the history of the term digitalization originates in the 17th century, being attributed to the development of the system of binary numbers. According to other studies, the current social system recognizes digitalization when referring to a conversion and integration process, analog into digital, by using a minimum of two characteristics of 0 and 1 [75]. Specific to processes and workflows, the ability of digitalization in using the same content of data, the workflow implies transformation from analog to digital data [76]. On the opposite side, digital transformation consists of a significantly more complex process that implies thinking and structuring. Within a business and/or organizational framework, digital transformation is used when solving problems in a creative way, by realigning both technology and business models to arise with fundamental changes by adapting the existing technologies [77]. According to previous research [78], digitalization does not treat old technologies and processes as a problem, but the starting point is a problem that, with the help of existing tools, may be solved through new thinking patterns. 

When teaching within organizations, the methodology approach suggests numerous techniques used in accordance to the size, industry, education degree and economic prospects of the business. Although, despite the array of methods used, the vast majority of the courses are provided through online supplemented materials. Within the given conditions, most of the workers within organizations expect trainers, leaders and instructors to provide information by using new media. According to a study performed in 2016 [79], 85% of the respondents expect their trainers to try out new development methods by using digital learning media, while 93% subscribe for a preference of a digital-analog structure. A similar study that was previously performed [80] shows that 83% of the respondents expect trainers to deliver by using new media, while 90% of them considered welcome the digital devices as instruments used for learning during at work. Moreover, the same study emphasizes that 71% of 10 respondents consider that a trainer/teacher/leader should rely on their own experiences and expertise when delivering information and methodology to others, by selecting digital media components to be recommendable for use. 

### 1.8. Digitalization and Workforce Sustainability

For the organizational market landscape, digitalization and sustainability are considered to be two of the most powerful influences of the 21st century. 

Digitalization in the context of organizational learning provides information with regard to teaching and learning workforce expectations, including the degree of digital media usage, individual manager/trainer’s expertise, online teaching, IT skills and the use of (personal) digital devices. 

The convergence of the business digitalization and sustainability concept in organizations and social contexts alike confer leaders and managers opportunities and challenges that often subscribe to organizational boundaries. Since digitalization is expected to create social boundaries and determine organizations to confront economic realities, sustainability intervenes as an anticipator of unintended side effects that enables workforce to properly cope with them. 

Digitalization is considered to be a driver to achieve sustainability within an organization and used by managers and leaders to optimize processes and workflows. There are many companies that still have an inclination of using traditional informing and training ways, such as paper, when completing tasks, despite digital tools and procedures. In order for a business to reach a sustainable workforce, besides using their experience and knowledge, leaders should also consider digital tools and procedures for workforce education.

**Figure 1:**
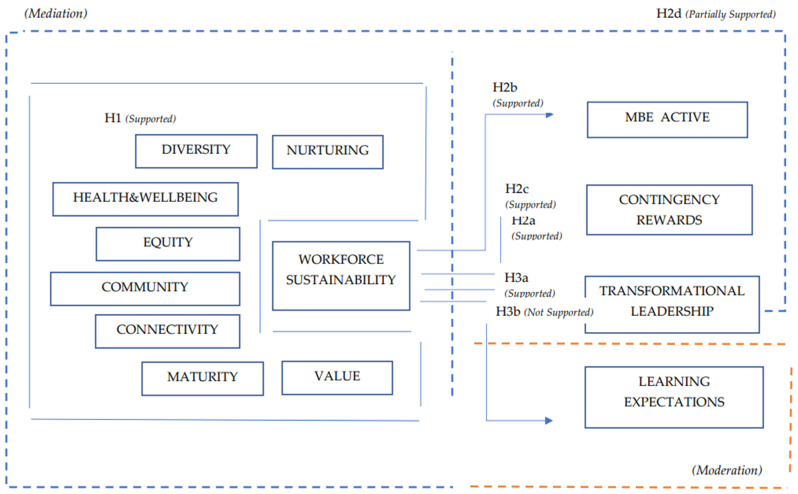
Final model of workforce sustainability, leadership and learning expectations. Source: authors’ development.

Digitalization is also useful for work actions traceability and transparency, encouraging a decrease in material and energy consumption, an optimization of materials usage, and implementing tracker user assets in order to reach a minimized supply chain issue array (see Figure 1). Given the current arguments, the following hypothesis arises:

**H3a.**  *Workforce sustainability is positively related to digitalization through learning and workforce expectations*.

**H3b.**  *The relation between workforce sustainability and digitalization through learning and workforce expectations is mediated by transformational leadership*.

## 2. Materials and Methods

The current research model implies a mixed qualitative-quantitative methodology; given the subject’s genuineness, based on the research question, performing a literature review by following the SR research flow in regard with “workforce sustainability” seemed the most appropriate method. Since review of the Scopus database only conferred a small number of available titles (one) in regard with workforce sustainability, a review of multi-industry resources was included, thus allowing an examination of publications in regard with the studied subject.

Further, a qualitative research instrument was designed, focusing on the workforce sustainability [4] in the context of transformational and transactional leadership [81] and digitalization through teaching and learning [80]. After performing a pilot study (resulting from applying the questionnaire via Google Docs) on a wide range of communication platforms specific to legally bounded active workforce (employees) in Romania, final data was gathered. We highlight the fact that the link for the Google Docs file was shared only within online active working groups, therefore the respondents only subscribe to the active workforce workgroups. By using SmartPLS (v.4.0.0.) the database will be tested in regard to the internal consistency and reliability of the new proposed research instrument (WS), followed by a SEM analysis and ending with a moderation analysis in regard to the entire instrument. 

The reason behind choosing to apply the research instrument to a mixed pool of workers resides in the novelty of the applied questionnaire. The current research is therefore to be considered a test-run of the workforce sustainability questionnaire; the intention of the authors is to continue to receive answers from working population, extent the database and perform a sectorial analysis that may bring into light workforce sustainability differences and similarities. 

For the correct development of the current research, a first analysis was performed in the form of a pilot study, where the Google Forms survey link was distributed online via various communication platforms. After data gathering, 34 answers were selected for interpretation. As previously suggested by literature [82,83,84,85], the accepted method that allows researchers to make changes around the initial methodological approach, data gathering, administration and interpretation, as well as the study design, is the pilot study, that also provides researchers with the possibility of preventing and/or avoiding shortcomings. As for the pilot study developed for the compiling of the current research, after a thorough analysis, data revealed that no misunderstandings or item miswording was in order, therefore the entire instrument was considered to be relevant for further developments. 

### 2.1. Participants and Procedure

A total number of 505 participants, aged between 14 and 43 years old agreed to participate to the current study. The reasons behind choosing the age interval 14–23, resides within the working force realities in the field. As for our case, there was no underaged respondent (18 ani). We should mention that the reason behind inserting the age interval subscribing to 14–23 as part of the current research resides within the Romanian legislation, namely the HG 75/2015 [86], which allows minors to be part of paid contracts, with the express agreement of their tutors/legal supervisors. After a careful analysis of the contents of the gathered data, authors removed a number of 42 incompletely or irregular filled in questionnaires. Out of the 463 correctly filled in questionnaires, data showed that more than 50% of the respondents were aged between 14 and 23 years old (260 respondents), while 21% subscribe to the 24–33 years old category, while the least representative group is the one of 43+ years old (with 11.9% of the total participants). As for the gender of the respondents, the percent is evenly distributed, 52.5% female and 47.5% male. As for the fields they activate in, out of a variety of answers, the predominant domains regard IT, human resources, public administration, medicine, marketing, tourism, physics, sociology and services. 

### 2.2. Measures 

As for the measures of the current study, a 64-item questionnaire was distributed online by using the Google Forms platform. The applied instrument was reduced in scale, by reconfiguring the item number that regard transformational leadership, MBE active and contingent rewards, as for the respondents not to fill in and leaving blank; additionally, a setting for “required answer” was also established. 

The instrument used for the current research consists of three parts: initially, a demographical status section was considered; the second part implied studying the five dimensions that regard on one side workforce sustainability (WS) and four other dimensions, two dedicated for studying leadership (as one component dedicated to transformational leadership and two components specific to transactional leadership, namely contingency reward and MBE active), and a third dimension that studies the digital transformation through its component of digital teaching and learning. As for clarifying the design of the current research, an example of questionnaire latent variables and items are presented within Table 2.

For instrument construction, the researchers selected a 7-Point Likert Scale, despite the frequency of the 5-Point Likert Scale. The reasons behind the current decision are that, according to literature, the data accuracy decreases when using a scale dropping below 5 but increases its quality when using a scale over 7. By considering the current findings, the authors considered more adequate the usage of a 7-Point Likert Scale, as being more suitable for online usage and electronic distributing. Research shows that a symmetric 7-Point Likert Scale is perceived more friendly by online respondents, since it provides an increased number of options for selecting, and therefore increasing the possibility for the respondent to express its option more accurately; the current method has the considerations for meeting the objective reality and thus appalling for the respondents’ reason faculties. 

#### 2.2.1. Workforce Sustainability Measures

As for regarding study variables, workforce sustainability (WS) comprises eight different dimensions [4] as follows: nurturing (six items), diversity (six items), equity (five items), health and wellbeing (five items), connectivity (five items), community (four items), value (nine items) and maturity (six items). Participants were asked to respond with regard to the items by using a 7-point Likert scale, ranging from 1–7 as (totally disagree—totally agree). The average internal consistency for WS is 0.97.

#### 2.2.2. Transformational Leadership Measures

The transformational leadership component was formed by considering all the four initially introduced within the literature dimensions [81], such as idealized influence (attributes and behaviors), inspirational motivation, intellectual stimulation and individual consideration, as presented within the 45 item MLQ (the 5X form) questionnaire. For the purpose of the current study, the instrument was adapted from the original framework of using a 5-Point Likert scale out of five items, into a 7-Point Likert Scale as previously mentioned, from 1–7 as (totally disagree—totally agree). The TL internal consistency is 0.90.

#### 2.2.3. Transactional Leadership Measures

Transactional leadership behavior was measured by using six items originating from the MLQ 5x form [81].

The two dimensions related to transactional leadership, MBE active and contingency rewards, consist of three items each; in order to provide the answers, the respondents could use the rating of a 7-Point Likert Scale, from 1–7 as (totally disagree—totally agree). As for the average internal consistency, contingent reward (CR) registered a value of 0.84 while MBEA averaged 0.82. 

#### 2.2.4. Digitalization through Teaching and Learning Measure

In the case of analyzing the digitalization component, data was provided by taking into consideration instead of an initially proposed 5-Point Likert Scale [80], a 7-Point Likert Scale, from 1–7 as (totally disagree—totally agree), split between a number of six items. The internal consistency of the proposed dimension was 0.85. 

After revising the relevant literature, results show that the role of the parameter estimates along with the control variables is considerable for developing new research. Authors consider it important to highlight the fact that for the development of the current study, no control variables were used; the reason behind the current choice is reducing the possibility of results to provide a reduced statistical power and degrees of freedom. 

### 2.3. The Analysis Strategy

As for assessing the relationship among the five constructs and nine subconstructs, for the correct development of the current research, the SmartPLS (v.4.0.0) software was used, with the purpose of initially testing the internal consistency and reliability of the new proposed research instrument (WS), followed by a SEM analysis and ending with a moderation analysis. The research objective suited by the authors was to provide, analyze and assess organizational workforce sustainability, in a context of emerging leadership and advancing digitalization. The increased complexity of the theoretical model required a SEM analysis that provides a better understanding the intricacies of the existing relations among the specified variables. 

SmartPLS is recommended by literature in cases when the model includes formative constructs (at least one), by applying the partial least squares algorithm [87,88]. The software is based on partial least squares, thus generating two models: the outer model provides data in regard with the infrastructure of the observable variables (yielded to the latent variables), while the inner model provides research with a structural model relating with the proposed model latent variables to another latent variables. 

Initially, researchers considered important to provide data by testing the outer model and refer to the reliability and validity of the latent variables; moreover, as previous studies state [89], testing the inner model and using the path coefficients, data is being provided in regard to the outer model constructs connections.

### 2.4. Methods Setting and Sample

The current study is based on the prior literature findings practice and pragmatism, by using a voluntary response convenience sampling method. Moreover, data gathering used the online Google Forms platform, and the WS developed instrument was presented to various organizational representatives; in order to reach the respondents, an increased number of online applications and electronic communication means were used. 

As for respecting the General Data Protection Regulation (GDPR), when accessing the questionnaire, the respondents were informed that no personal data was to be retained or requested; a strict confidentiality of the answers was guaranteed, along with the fact that all data will only be used for the purpose of academic research.

While using a type of non-random sampling [90], the current research started with running a pilot test for the current survey, followed by generating hypotheses that followed to be tested. As previously mentioned, the ways used to draw the current convenience sample included online via social media posting, along with sharing the survey through pre-existing groups from different organizations. The survey starts with identifying and defining the variables to the target audience, individuals in Romania that currently develop employment activities within an organization. As explained within the Methodology section, data gathered from individuals that did not develop activities under an employment (or similar) framework, was not considered for further analysis. 

The questionnaire design is intended to provide at first another perspective on the insights of the workforce sustainability concept by designing an assessment instrument based on the eight dimensions proposed [52]. Furthermore, the research continues by assessing if there is a link among the workforce sustainability (WS) as currently defined and three leadership components, as transformational leadership, and two other elements specific to transactional leadership, MBE active and contingency rewards. Researchers also considered another link that could occur in regard to WS, by adding a component of digital transformation, namely expectations of teaching and learning. 

When considering the case when respondents agreed with filling in the survey, they were provided with the information that no type of compensation was to be in order. Since the survey was distributed online and information was gathered based on convenience sampling, the response rate is considered to be low, being developed within a two-month interval, and receiving a number of 505 responses. 

## 3. Results

According to literature [91,92], when assessing a new instrument, a series of actions need to be performed. A first action regards checking the collinearity among the given constructs; according to the current analysis, since all the factors are reflective, a run of consistent PLS SEM algorithm, path, standardized was in order. VIF (variance inflation factors) values are designed to quantify the severity of the collinearity among the proposed model indicators; as for the current results, VIF takes values from 1.21–3.25, which according to previous authors [87] subscribe to the threshold of <4.0 that indicate that a given model does not have a problem in regard with multicollinearity.

The standardized path coefficients of the current model subscribe to the range 0.1–0.8, thus subscribing to the requirement of being <|1| therefore confirming that the current model does not have multicollinearity issues (in cases where path coefficients subscribe to values greater than |1|.

According to previous studies recommendations [93], the assessment of the structure model must continue with a bootstrapping procedure, to assess the significance of the path coefficients and R^2^. 

As numerous authors consider [94,95], the R^2^ values of >0.67 are considered to be substantial, >0.33 are considered as moderate, while values >0.19 are considered to be weak. There are authors that suggest another classification [46,96,97] independent to the field of study. According to their views, correlations are weak (0.00–0.29), low (0.3–0.49), moderate (0.5–0.69), strong (0.7–0.89) and very strong (0.9–1.00). Literature validated another classification only reliable in the field of behavioral sciences [98], where the effect size “r” is small (r = 0.10), medium (r = 0.3) and large (r = 0.5). According to literature [99], the R^2^ values should be comprised of values ≥0.1 as for the particular constructs explained variance to be considered as adequate. As for interpreting R^2^ of latent variables in PLS SEM, it is considered that R^2^ < 0.25 are very weak, 0.25 ≤ R^2^ are weak, 0.5 ≤ R^2^ < 0.75 are moderate, while R^2^ ≥ 0.75 are substantial [100,101,102]. Therefore, the R^2^ values for contingency reward and transformational leadership are substantial [103], while digital learning expectations and MBE active are weak to moderate. 

In order to validate the proposed reflective model, outer loadings need to be considered first. Outer loadings are required to have values >0.7, but if the dimension averages out 0.7, it is accepted for all loadings to be kept. Moreover, factor loadings <0.5 are considered to be low, thus following to be deleted [87]. 

Key literature in regard with the usage and reporting of the SmartPLS software suggests that data should be assessed and instead of reporting values of “Cornbach’s Alpha” for the instrument reliability analysis, the “Rho A” values should be used [104,105,106]. As Table 3 shows, the “Rho_A” Values for all the selected variables subscribe to the interval 0.79–0.97, thus confirming the given construct to be a reliable composite. 

Further, in order to analyze the convergent validity of the construct, the AVE values were considered; according to literature [107,108], the AVE values must comply with the criteria of averaging >0.5; if the given condition cannot be satisfied, indicators that have outer loadings <0.4 need to be dropped, while indicators that subscribe to the interval 0.4–0.7 can be retained, under the condition of not affecting the CR and AVE values [109,110]. As for the current study results, according to the initial construct reliability and validity table, all the selected factors subscribe to the parameters for having convergent validity, but according to the Average Variance Extracted (AVE) criteria, the diversity dimension = 0.492, and WS = 0.455 have values less than 0.05; after a careful consideration of the factor loadings of the diversity dimension, DIV3 = 0.598 and DIV4 = 0.599 from the were eliminated, resulting into an increase of the AVE values for both diversity (=0.549) and WS (=0.465) AVE values (see Table 3). Despite the AVE value for WS that has a value of less than 0.05 (=0.455), according to the CR values that can be used for measuring convergent validity (CR for WS = 0.974), it is considered to have sufficient convergence [111].

For the purpose of satisfying all literature views with regard to reporting indicators when performing the analysis of construct reliability and validity, and since the WS construct is exposed for the first time for external views and assessment, the authors decided to also report Cronbach’s Alpha and composite reliability values; as the authors suggest, values >0.7 are considered to be very good for both indicators; therefore, the Cronbach’s Alpha subscribes to the interval 0.78–0.97, while CR reports values between 0.79–0.97, therefore the model subscribes for being satisfactory and has convergent validity. 

As for the Standardized Root Mean Square Residual (SRMR) values, for both saturated and estimated models, SRMR = 0.067 subscribes to the range of <0.1o provided by literature [95] and even to a more conservative version of <0.08 [112] therefore providing a good fit for the current model. 

The SmartPLS provides the authors with the possibility of designing the measurement model and determine if the model’s indicators are best specified as formative or reflective. The SmartPLS software provides default models with a reflective design for all the models latent variables, reason for which a Confirmatory Tetrad Analysis (CTA-PLS) was considered as necessary; it is important to specify the fact that this analysis is only allowed to be performed in cases where latent variables comprise at least four associated indicators. For the current model, all the latent variables were tested, except for CR and MBEA. The results show that all the latent variables are best considered by reflective; the current results are best indicated by the *p*-values, since according to literature, for *p* < 0.05 (considered to be the threshold for significative values), the result is to be best formative, while for *p*-values < 0.05 (considered as not significative), it is best to be considered as reflective. Moreover, literature suggests another condition to be considered when variables with a large number of items, by introducing a general threshold value of 80% for the *p*-values; the interpretation of the current condition assumes that if 80% of the *p*-values are significant, the model is considered to be formative, while if 80% of the *p*-values are non-significant, then the model is considered to be reflective. 

In order to analyze if the data is affected by uncovered unobserved heterogeneity for the inner model, the authors provided a FIMIX analysis (FIMIX-PLS) considered to be a latent class method for segmentation [113]; the method is used in order to estimate the probabilities of hidden segments altogether with estimates of path coefficients specific to the hidden segments (in case they exist). As numerous authors claim [67,114], empirical research is not rarely affected by hidden heterogeneity, whose external sources cannot be controlled of known a priori. Moreover, it is not possible for researchers to know if hidden heterogeneity is affecting their estimations, therefore latent class techniques and approaches as FIMIX-PLS may be applied [113,114,115]. According to numerous previous authors [116,117,118], researchers should use as a routine technique the evaluation of unobserved heterogeneity for the data. 

For the purpose of determining the number of segments to extract, as previous literature claims [119], a minimum sample size is required to be computed; by assuming an effect size of 0.15 and an 80% power level allowed researchers to extract a maximum number of six segments. 

Determining the number of segments that need to be retained from the data implies assessing the minimum values of certain information criterion as Akaike’s information criterion (AIC) modified with factor 3 [25,107], consistent AIC, also known as CAIC [120] and another measure that helps of finding how well are the segments separated, by using entropy-based measures such as the normed entropy statistics (EN). As other authors suggest [121], EN is used in order to indicate if a partition is reliable or not; for this reason, EN that ranges between 0 and 1 provides data as for a higher value to indicate a higher quality partition. As previously suggested [122], EN > 0.50 allow researchers to cut data into a predetermined segments number. 

The results of the fit indices for the one-to six segments present an ambiguous scenery (see Table 4). As mentioned previously [123], when there is the case when AIC 3 and CAIC indicate towards the same segment, then results are likely to show the corresponding number of segments for the given database. As for the current results, AIC3 indicates a five-segment solution, while the CAIC indicates a two-segment solution. Moreover, numerous authors suggest that AIC4 and BIC (Bayesian information criteria) register have a good performance when are used for determining the segments number by using the SmartPLS Software [124]. For the current case, both criteria, AIC4 and CAIC point towards a two-segment solution which according to literature [125] is densely clustered if taking into consideration the EN Criteria. Moreover, as a study form 2020 shows [124], a solution with two segments represents the minimum requirement for sample size segments. However, MDL5 (minimum description length with factor 5) points towards a 1-segment solution. As previously suggested [126], MDL5 has a pronounced tendency to present solutions with an underestimate number of segments, therefore for the current research, performing analysis for a six-segment solution was a right choice. Considered together, the indices of the current analysis ambiguously point to a specific segment solution, since AIC3 points to a five-segment solution, while CAIC, AIC4 and BIC point towards a two-segment solution, hence the MDL5 which points towards a one segment solution. Previous literature shows that it was performed a study with a five-segment solution and observing a similar behavior [127], therefore it is not wrong to assume that unobserved heterogeneity for the current dataset has not reached a critical level, therefore supporting the results of the entire dataset. Considering the current results in regard to the data unobserved heterogeneity, sufficient evidence is provided in regard with the results robustness [123]. 

As FIMIX is only an exploratory assessment tool, all the criteria information performance, including EN, are fallible only if data has low levels of collinearity specific to the assessed model [115]. Moreover, [119,128] as claimed before, all the segments should be small enough for providing parsimony and manageability, but also to be sufficiently large as to be given strategic attention.

**Figure 2:**
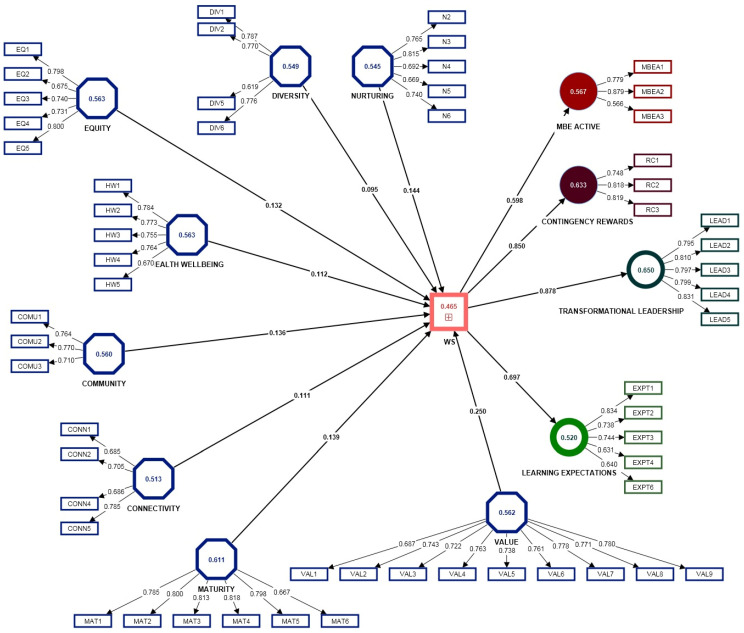
Outer factor loadings model. Source: authors’ calculation with SmartPLS (v. 4.0.0) software.

Results show significant differences between the importance of certain predictors based on which segment is under analysis (see Table 4; Figure 2).

The research continued by considering the serial and specific indirect effects (mediation); as it is easy to observe, the construct presents direct effects from each of the five dimensions specific to the WS (the dependent variable), and also mediating effects through WS in regard with three leadership components (MBE active, contingency rewards and transformational leadership), but also a digital component that studies teaching and learning expectations. As can be seen from Table 5, the results show all implied specific indirect effects, including serial mediation that start with the left most independent variable. The results show the case where *p* values < 0.05 (implying significance) as in most of the cases, except connectivity, maturity, nurturing, equity, health and wellbeing and diversity → WS → transformational leadership, where there is no implied indirect effect for the current model. 

**Figure 3:**
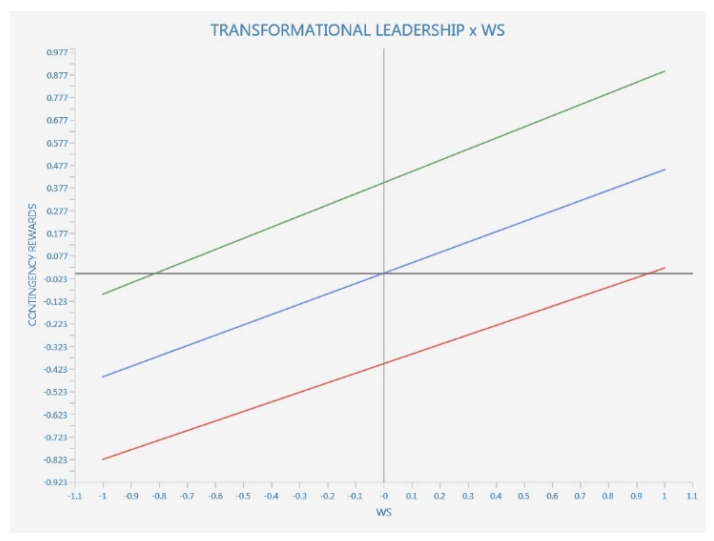
Contingency Rewards and Workforce Sustainability Simple slopes analysis. Source: authors’ calculation with SmartPLS (v. 4.0.0) software. The positive relationship between WS and contingency rewards is almost parallel, so there is no real moderating effect.

**Figure 4:**
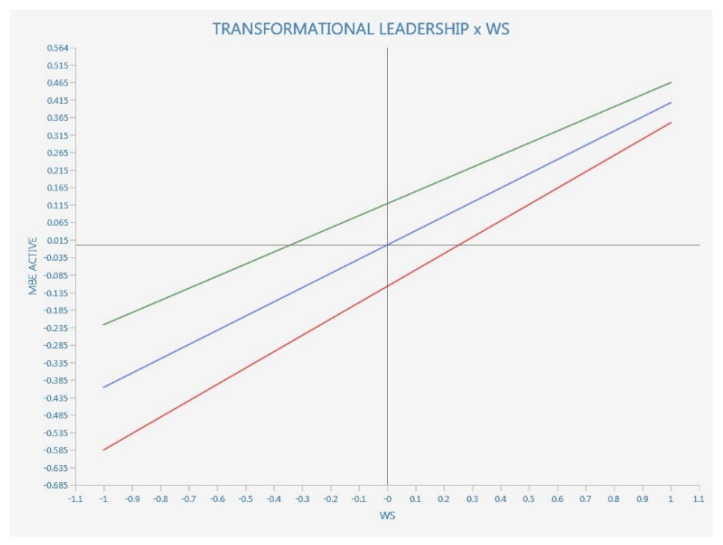
MBE Active and Workforce Sustainability simple slopes analysis. Source: authors’ calculation with SmartPLS (v. 4.0.0) software. The positive effect (slopes are bottom to top, left to right) of WS on MBE active is dampened (has a steeper slope) when there is less TL (is not amplified/strengthened by TL).

**Figure 5:**
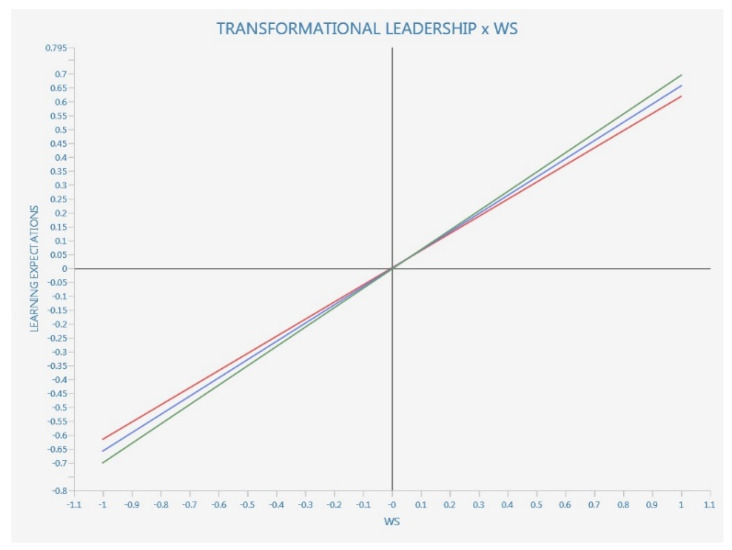
Learning Expectations and Workforce sustainability simple slopes analysis. Source: authors’ calculation with SmartPLS (v. 4.0.0) software. There is an inverse effect between TL and the positive relationship between WS and digital learning and education. Meanwhile, TL begins to have an inverse effect (the green line is with more TL, the red line is with less TL), meaning that the relationship between WS and digital learning and education, has a steeper (positive) slope when there is less TL. A positive effect (it is slipping bottom to top, left to right, and the mean—blue line is sloping upward) between WS and digital learning and education is increased by TL.

We also performed an interaction moderation with simple slopes plot where we considered to be a moderating variable the transformational leadership vs. all the other dependent and/or endogenous variables, including WS (see Figure 3, Figure 4 and Figure 5).

Since standard deviations are slipping bottom to top, left to right, we have a positive effect. The red line is with more transformational leadership, while the red line is with less transformational leadership. 

The positive effect has a parallel and not a steeper slope, so there is no moderation. 

**Simple slopes analysis**

**Figure 6:**
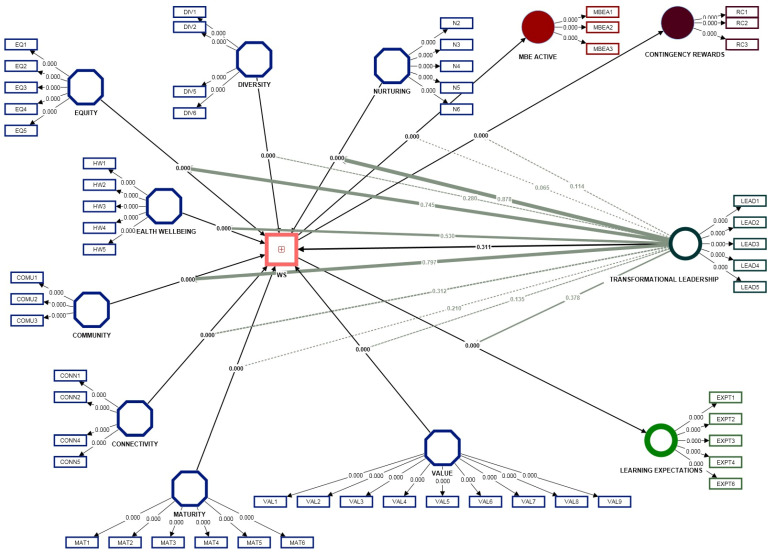
Leadership moderation with *p* values highlight with absolute values. Source: authors’ calculation with SmartPLS (v. 4.0.0) software.

As literature suggests, the simple slopes analysis provides data that is more reliable than the *p* values provided by the bootstrapped path coefficients from the moderation analysis (see Figure 6). 

According to Figure 3 results, TL show significant moderating values for three of the WS attributes, namely nurturing, equity and community, while the teaching and learning component of digitalization in relation to WS does not seem to be significatively moderated by TL.

### Hypothesis Testing

The first hypothesis suggests that workforce sustainability is positively related to eight individual attributes as nurturing, diversity, equity, health and wellbeing, community, connectivity, maturity and value.

We tested a model where paths from all the eight attributes to workforce sustainability, in order to examine the unique contribution of each attribute to the workforce sustainability. The path from community and connectivity to WS was 0.11, while diversity, equity and health and wellbeing to WS was 0.12, the paths from nurturing to WS was 0.13, maturity to WS was 0.14, and value to WS was 0.25. This means that H1 is supported. 

Furthermore, we tested the model as WS to be positively related to TL, MBEA and CR. The path analysis suggests that WS to TL was 0.87, WS to MBEA was 0.59 and WS to CR was 0.85, thus supporting H2a, H2b and H2c. 

Further, we tested whether the relationship between workforce sustainability and each of its eight attributes is moderated by transformational leadership. The results suggest significant values only in the case of nurturing, equity, community and health and wellbeing, therefore only partially supporting H2d. 

We also tested whether workforce sustainability is positively related to digitalization through learning and workforce expectations. The results suggest that 70% of workforce digitalization is explained by WS., therefore supporting H3a.

We finally assumed that the relation between workforce sustainability and digitalization through learning and workforce expectations is mediated by transformational leadership. The results show an insignificant value for the mediation relation between the two constructs, therefore H3b is not supported. 

## 4. Discussion

The current study had a threefold aim. First, the authors proposed and validated a new assessment tool for the workforce sustainability concept. Second, leadership as transformational and transactional (through CR and MBEA) was considered to be explained by workforce sustainability within an state and/or private organizational context. Further, authors examined how digital learning and workforce expectations interact with the workforce sustainability processes. Moreover, we examined the relationships by considering transformational leadership as a mediator between WS and its eight components and CR, MBEA and the digitalization component of teaching and learning. 

The uniqueness of the study derives from the fact that our proposal is the first of this kind to assess the subject of workforce sustainability through a specific qualitative tool, with multiple components that were assessed and tested within an organizational context. Moreover, after validating the construct, workforce sustainability was assessed in relation to leadership and digitalization components deriving from the organizational context. 

Given the lack of instruments that are able to provide organizations with valuable insights with regard to the sustainability of their own workforce, and the fact that the research instrument was applied online, the population of the current study reflects social and organizational realities that are accessible for the applicability and implementation of the current study. Further, we will now discuss the findings in detail. 

### 4.1. The Eight Attributes of Workforce Sustainability

In line with an earlier theory [4], we found that after testing the composite reliability and the variance of each of the eight attributes, they contribute to the sustainable workforce development within organizations. Such a validation confirms the utility of the tool within organizational environments, and also identifies potential areas of improvement for its design and components. Moreover, a supporting study should be enhanced as for examining any existing correlations among workforce sustainability within different organizations and /or industries (e.g., IT, medicine) and important performance indicators as the quality of work, individual productivity, regional or rural professional satisfaction [129] safety and individual and organizational performance. In the eventuality of performing such a study, the workforce sustainability tool could justify its importance and could gain interest within the business and social environments. 

### 4.2. Workforce Sustainability and Leadership

In the context of organizational sustainability, leadership, defined and assessed by [81], is a direct contributor of workforce maturity, by facilitating and enhancing individuals and groups communication and development skills. Moreover, leadership is at core of the ethnic, gender or racial diversity and inclusion within an organizational and/or social context. Assessed through a multitude of online and/or on-site tools and instruments, leadership, transactional and/or transactional, is capable of creating a framework to enhance workforce sustainability and mediate its attributes. Workforce sustainability has the property of reflecting the extent to which organizational employees accomplish and perform their duties, over a given framework and period of time; therefore, a sustainable workforce is based on the requirement of a continuous workforce development and intense individual cultivation, tasks that can only be performed by a healthy leadership organizational framework. We found that workforce sustainability is partially explained by the leadership components (transformational and transactional); this validation confirms once more the utility of the proposed model and identifies areas for further improvements. Further studies should include a fair assessment of the attributes, indicators and metrics specific to a controlled number of organizational environments, and an analysis could depart from an existing problem within their internal systems. Therefore, highly skilled and competent leaders should be provided with results, in order to maintain the requisite workers skills and improve and cultivate their competencies, with regularity. 

### 4.3. Workforce Sustainability and Digitalization

Digital tools and forms, along with mobile communication and interaction help organizations to reduce costs and increase performance. Scheduling, route planning, traceability are features that enhance an organization workforce flows, thus creating a framework for sustainable development. We showed that digitalization through learning and expectations are an important driver for workforce sustainability; moreover, the relationship between the two constructs is reluctantly moderated by leadership behaviors, thus creating an apport for the usage of digital tools and instruments within the change processes necessary for creating and favoring the sustainability of the workforce. Further developments should also consider including into the analysis further digitalization components and studying the degree to which a workforce sustainable organization subscribes to the usage of the Internet of Things (IoT) and the Internet of Everything (IoE) practices. 

### 4.4. Theoretical Implications

The current study’s contributions to literature count in a number of ways. Most importantly, it is the first to examine the influence of the eight attributes on workforce sustainability within an organizational context. Contextual studies are important since they bring us closer to the workers’ realities, since the respondents were only considered if currently employed, therefore, as other authors suggest, reducing the risk of recall bias [130]. Therefore, workforce sustainability measured under working or close to working hours may represent an increasingly accurate reflection of the organizational context, compared to a measure that happened at one point in time. 

Moreover, the current research represents one of the first of its kind that examines the influence of transformational and transactional leadership (via different components) on workforce sustainability. According to previous literature [5], transactional leaders cannot influence the work engagement of followers, but according to previous authors [131], to some extent, CR is stimulating followers work engagement and thus, influences the sustainable individual behavior at work. Transformational leadership on another side, augments the effects of transactional leadership; it has often been questioned whether transformational and transactional leadership behaviors [132] react differently in the context of a sustainable organization. We could not provide an answer to this question, but our study suggests that it is worthwhile to study the effect that leadership (as transformational and transactional) has on workforce sustainability, considering the eight attributes separately. 

As for the industries that workforce sustainability needs and can be enhanced and assessed, literature shows [133] that organizational change management when about projects or intensive training platforms, encounters the need to assess and create a sustainable flow of working hours and environments, as for improving patient management (in medicine), increase the training abilities (in IT) and/or develop sustainability pathways (in sociology).

Finally, the present study enhances the call for more research in the area of organizational digitalization and the underlying relationship with workforce sustainability and work outcomes [134]. We showed that digitalization through its component of learning and workforce expectations is directly linked with workforce sustainability, and that the mediating role of leadership does not intervene in the given context. Nonetheless, we contribute to both leadership and sustainability literature since we consider the positive affective and motivational outcomes considered by employees, and relate them to workforce sustainability, contrary to the organizational stress and burnout considered by the majority of the studies in the field [135]. 

### 4.5. Practical Implications

From the point of view of practical implications, the current study shows the importance of workforce sustainability and its eight components for the business environments and organizational contexts. Besides, when an organization has an “off-performance” and disengages or incoherently or mistakeably performs actions in one or more of the eight attributes, the entire organization’s workforce sustainability is at risk. A widespread of the current assessment workforce sustainability tool is expected to enrich a high number of industries [52], to develop and train its workers in order to result in a health and resilient organizational workforce environment. Our results confirm the current allegations and show that when considering workforce sustainability, both organizations and employees recognize the importance of the eight attributes in the workplace.

Since workers may not always be aware of the reasons under which a company acts or defends its interests, the assessment of leadership components under the same construct’s umbrella may prove to be useful in order to provide workers with feedback in regard to their actions and behaviors. The process is two-sided, since leaders could also take actions following the employee’s feedback. Research has shown that leadership (as transformational and transactional—MBEA and CR) has a direct positive effect in regard to workforce sustainability. According to previous research [136,137], leaders can be trained when performing within an organizational umbrella while performing their tasks. However, in the light of the current research, it is important for leaders not no use only transformational tools and behaviors, but to also associate conducts that derive from transactional contingent reward and management by exception. 

In the end, it is important to relate in regard with the practical applications of digitalization within the organizational learning processes, that lead to an increased performance for both organization and for its workers individually, but also for a particular organization’s workforce sustainability that may reflect attitudes and decisions of a particular business environment or even an industry. 

### 4.6. Limitations of the Study

Although the current research design has clear strengths, one must not consider it without limitations. The use of qualitative assessment tools has the potential to increase the risk of bias [46], but it may affect the variance [82]. Furthermore, we examined how workers are affected by the organizational policies they work within regarding the particular indicators of the eight dimensions, which is a private experience; according to previous research [138], bias for commonly used methods is rare and unlikely to occur [109] in a situation likely to be true for the context of the present study since the constructs show relationships that best qualify as moderate [110]. According to previous research suggestions [82], by ensuring the participants anonymity, we reduced the effects of common method bias. 

Since we did not include transactional leadership as MBE passive within the study, it induces the thought of another possible limitation. Among the aims of the study one can count the importance of the differentiation of different transactional leadership components, since literature shows that not always all the components relate positively to organizational features as (workforce) sustainability. The MBE passive was not included since according to literature [67], leaders use MBE passive when they find it difficult to monitor mistakes among large numbers of workforce. Considering the tendency for group and teamwork within companies that act on the considered geographical region, we assumed that leaders predominantly use MBE active instead of MBE passive. Moreover, both MBE active and passive are expected to gather similar results, with a difference of the time for the leader’s intervention. For future research, instead of TL and two components of transactional leadership, MBE passive could also be included within the design on the construct. 

A specific sample and context may be needed for future research, in order to assess through specific instruments, the degree of digitalization of an organization or an industry, including workforce expectations for teaching and learning. 

Since the study only considered 463 responses, the generalizability of the findings may be affected. The results of the current study are in line with the hypotheses that derived from the available theoretical background; despite this fact, results need to be subject for replication within higher number samples of employees, working under similar at first, and different conditions further. 

Within the current study the authors only centered on the reliability and validity of the workforce engagement construct, and the bipolar relations with leadership (transformational and transactional) and the digitalization of the teaching and learning processes. We showed that the workforce sustainability assessment tool is stable and provides a working framework for organizational internal and industry further analysis. Nevertheless, it is of utmost importance study the leadership and digitalization components in relation to a specific workforce sustainability framework, in order to be able to answer to the question of under which circumstances an organization workforce sustainability is enhanced by leadership and digital learning. As an example, according to earlier literature [139], leaders have a lower influence on the work environment when workers make use of their own material or online resources. Moreover, MBE active is highly effective within work environments with high-risk professions, therefore influencing workforce sustainability results on an organizational, societal or professional level [140]. 

## 5. Conclusions

Contributions to literature of the current research count a number of specific ways. First, to our knowledge, this is the first research that provides a valid tool for assessing organizational (public and/or private) workforce sustainability. Moreover, it is the first study to assess the impact that transformational and transactional leadership (as CR and MBEA), along with a digitalization component of teaching and learning workforce expectations on workforce sustainability. We showed that all the leadership components and digital learning and expectations component are positively related to workforce sustainability. Moreover, we assessed the mediating role of transformational leadership in regard to the eight attributes of workforce sustainability, but also on the leadership and digitalization components. The results show that transformational leadership is a mediator for four of the workforce sustainability components; it also showed that respondents make a clear distinction between transformational and transactional leadership behaviors, and that digital learning and expectations are only related to workforce sustainability, since no mediating role of TL is significant. To conclude, the current study shows that leadership and digitalization must be considered when an organization aims for enhancing a sustainable workforce. 

## Figures and Tables

**Table 2:** Example of dimensions and items specific to the research instrument.

Instrument Dimensions		Items Example	Source
Workforce Sustainability	Nurturing	At my workplace I often receive appreciations about my performance.	Authors development based on [4]
	Diversity	My company has clear policies that foster diversity and inclusion in the workplace.	
	Health and Wellbeing	In the company where I work, a security policy is observed that encourages zero work incidents.	
	Equity	At my workplace, an attitude of equality, fairness and non-discrimination is promoted.	
	Community	Within the organization, participation in social events organized internally is encouraged.	
	Connectivity	Within the company where I work, the involvement of employees in the decision-making process is encouraged.	
	Maturity	Within the company I work for, the development of leadership and communication skills are encouraged.	
	Value	Within the organization where I work, a policy of employment contracts and long-term employee involvement is respected.	
Mbe Active		Often my leader has focused his attention on irregularities, mistakes, exceptions and deviations from standards.	Literature adapted [81]
Contingency Rewards		My leader discusses in specific terms who is responsible for achieving the performance targets.	Literature adapted [81]
Transformational Leadership		Often my leader speaks enthusiastically about what needs to be achieved.	Literature adapted [81]
Learning Expectations		It should be allowed to use smart phones or tablets to study/ work when they are in the organization.	Literature adapted [80]

Source: authors’ development.

**Table 3:** Construct reliability and validity.

	Cronbach’s Alpha	Rho_a	Composite Reliability (rho_c)	Average Variance Extracted (AVE)
Community	0.792	0.794	0.792	0.56
Connectivity	0.808	0.811	0.808	0.513
Contingency Rewards	0.838	0.84	0.838	0.633
Diversity	0.827	0.835	0.829	0.549
Equity	0.866	0.868	0.865	0.563
Health Wellbeing	0.865	0.867	0.865	0.563
Learning Expectations	0.843	0.851	0.843	0.52
Maturity	0.903	0.906	0.904	0.611
MBE Active	0.789	0.821	0.792	0.567
Nurturing	0.856	0.86	0.856	0.545
Transformational Leadership	0.903	0.903	0.903	0.65
Value	0.92	0.921	0.92	0.562
WS	0.974	0.974	0.974	0.465

Source: authors’ calculation with SmartPLS (v. 4.0.0) software.

**Table 4:** FIMIX analysis and results.

	Segment 1	Segment 2	Segment 3	Segment 4	Segment 5	Segment 6
AIC3 (modified AIC with Factor 3)	1648.064	1377.753	1363.885	1365.331	1355.135	1377.816
CAIC (consistent AIC)	1718.624	1523.024	1583.867	1660.023	1724.539	1821.93
EN (normed entropy statistic)	0	0.624	0.695	0.57	0.625	0.589
SUMMED FIT	3366.688	2900.777	2947.752	3025.354	3079.674	3199.746

Source: authors’ calculation with SmartPLS (v. 4.0.0) software.

**Table 5:** Serial and specific indirect effects.

	Original Sample (O)	Sample Mean (M)	Standard Deviation (STDEV)	T Statistics (|O/STDEV|)	*p*-Values
Diversity → WS → Contingency Rewards	0.081	0.081	0.004	18.903	0
Connectivity → WS → MBE Active	0.058	0.058	0.005	11.657	0
Equity → WS → MBE Active	0.075	0.075	0.006	11.857	0
Health Wellbeing → WS → Learning Expectations	0.082	0.083	0.007	12.015	0
Nurturing → WS → MBE Active	0.075	0.075	0.006	12.501	0
Connectivity → WS → Transformational Leadership	0.078	0.06	0.105	0.737	0.461
Maturity → WS → Learning Expectations	0.108	0.108	0.007	16.105	0
Nurturing → WS → Learning Expectations	0.089	0.089	0.006	13.762	0
Diversity → WS → MBE Active	0.057	0.057	0.005	11.544	0
Maturity → WS → Contingency Rewards	0.131	0.131	0.006	22.691	0
Diversity → WS → Learning Expectations	0.067	0.067	0.005	13.332	0
Health Wellbeing → WS → MBE Active	0.07	0.07	0.005	13.021	0
Maturity → WS → Transformational Leadership	0.123	0.095	0.167	0.738	0.46
Value → WS → MBE Active	0.139	0.139	0.011	12.96	0
Nurturing → WS → Transformational Leadership	0.101	0.078	0.137	0.737	0.461
community → ws → contingency rewards	0.082	0.082	0.004	21.706	0
Health Wellbeing → WS → Contingency Rewards	0.1	0.1	0.004	22.252	0
Maturity → WS → MBE Active	0.091	0.091	0.007	12.707	0
Community → WS → Learning Expectations	0.068	0.068	0.005	13.294	0
Equity → WS → Transformational Leadership	0.101	0.079	0.138	0.735	0.462
Health Wellbeing → WS → Transformational Leadership	0.094	0.073	0.128	0.735	0.463
Equity → ws → Learning Expectations	0.089	0.089	0.006	14.008	0
Community → WS → MBE Active	0.057	0.057	0.005	12.489	0
Community → WS → Transformational Leadership	0.077	0.059	0.104	0.74	0.459
Value → WS → Transformational Leadership	0.187	0.144	0.254	0.738	0.461
Value → WS → Contingency Rewards	0.2	0.2	0.008	26.47	0
Value → WS → Learning Expectations	0.165	0.165	0.013	12.93	0
Diversity → WS → Transformational Leadership	0.076	0.059	0.104	0.736	0.462
Connectivity → WS → Learning Expectations	0.068	0.068	0.005	12.706	0
Equity → WS → Contingency Rewards	0.108	0.108	0.005	20.7	0
Nurturing → WS → Contingency Rewards	0.107	0.107	0.005	22.496	0
Connectivity → WS → Contingency Rewards	0.083	0.083	0.004	19.657	0

Source: authors’ calculation with SmartPLS (v. 4.0.0) software.

## Data Availability

Not applicable.
